# Sanitary Administration in America

**Published:** 1897-09-18

**Authors:** 


### SANITARY ADMINISTRATION IN AMERICA.

One great characteristic of American sanitary administration is the tendency shown by the authorities, when once a fact in medical science is recognised and accepted, to carry to their logical extremity any measures founded upon it. For how many years have we not recognised that diphtheria is due to a microbe? Yet how far have we gone in London in providing any means by which a bacteriological diagnosis may be arrived at? We have hardly moved a step. One or two vestries do something, and, of course, an investigation is made of the cases at the hospitals; but, so far as London as a whole is concerned, we might as well know nothing. No sooner, however, was it recognised in New York that the presence or absence of the bacillus was a point of importance, than a complete system of bacteriological diagnosis was put freely at the disposal of all physicians practising in the city.

Then in regard to the serum treatment what do we do? Nothing, except in connection with the hospitals, notwithstanding all that has been done to prove the efficacy of the treatment. In New York, however, the directors of the Board of Health laboratories at once undertook the preparation of the serum; they now distribute it free to all public institutions, they keep it on sale at a hundred depôts, where also it can be obtained free for patients unable to pay for it, and not only so, but a special corps of medical inspectors is maintained for the administration of anti-toxin; and, on request, one of these inspectors will visit a person suffering from diphtheria, in any part of the city, day or night, and administer the serum under the supervision of the attending physician. He will also immunise the rest of the family if they have been exposed to the disease.

Diagnosis is a well recognised stumbling-block, in the face of which, however, we fold our hands and content ourselves with issuing a report stating in how many cases admitted as diphtheria the diagnosis has turned out to be incorrect. To prevent this great evil of admitting patients into infectious wards we do nothing officially. The Medical Officer of Health does not even visit the case. In New York things are very different; not only is the bacteriological test freely employed, but special officers, called diagnosticians, two of whom are on duty day and night, are employed to give expert assistance in the clinical diagnosis of contagious diseases.

Turning now to tuberculosis, we find from Dr. Hermann Biggs' address at Montreal that full use is being made of the knowledge we possess in regard to its bacillary origin. The full courage of scientific conviction seems, he says, to have been generally lacking among public officers in dealing with this disease. It is now generally admitted that tuberculosis is infectious and communicable, and is the most fatal disease to which the human race is subject; yet, as a rule, no effective measures, or no measures at all, have been adopted by sanitary authorities with relation to it.

These opinions are no doubt held as widely in England as in America. But compare our *laisser faire* methods with what is done in New York to limit the spread of the disease. In 1893 notification of all cases of pulmonary tuberculosis occurring in public institutions was required, and physicians were requested to report cases in private practice. Arrangements were also made for the bacteriological examination of sputum by the officers of the board; all reported cases in tenement-houses, lodging-houses, hotels, and boarding-houses were inspected, and the patients and their families were instructed as to the means to be taken for the prevention of the disease; all premises in which death from tuberculosis had occurred were inspected; orders were issued upon the owners of apartments which had been occupied by consumptives, requiring that they should be thoroughly renovated by cleansing, painting, &c., and placards were attached to the doors to prevent their re-occupation before the order for renovation had been complied with. Under the resolutions by virtue of which these measures were enforced, 4,166 cases of tuberculosis were reported in 1894, 5,818 in 1895, and 8,334 in 1895. So far as was possible, all of these cases, except those in private houses, were visited, or the premises where they had lived were inspected; and, in addition, the premises occupied by persons dying from tuberculosis (numbering each yearly 6,000) were inspected, and such action taken as was considered possible and desirable. Altogether, the premises and cases coming under observation during these three years numbered more than 35,000. This year a further step has been taken by declaring tuberculosis to be an infectious and communicable disease, dangerous to the public health, and requiring the notification of all cases occurring in the city.

Again, the knowledge that tuberculosis can be transmitted by milk—knowledge which in England has remained practically sterile—is at once acted on in New York. The sale of milk is absolutely prohibited without a permit from the health department, which is not issued until the dealer has furnished information as to the source from which the milk is obtained, the number of animals, the character of the food supply, and the sanitary conditions surrounding the dairy. These permits can be revoked at any time, and thus the whole milk trade is under the absolute control of the department; and still further to ensure freedom from infection all milch cows in New York city are now being tested by tuberculin, and those found to be diseased are killed.

Year after year sanitarians are becoming more and more convinced of the importance of school influence in the dissemination of infectious diseases. Yet, after all the money this country spends on education, it hesitates to pay for skilled inspection of the children who are compulsorily herded together in public schools and play-grounds.

In New York, the evil being recognised, the remedy is applied. Early in this year 150 medical school inspectors were appointed by the Health Board to examine daily, at the opening of the public schools, all the children who are set apart by the respective class-room teachers as not appearing to be entirely well. Every pupil found to be suffering from any form of general contagious disease, or any contagious disease of the eye or parasitic disease of the skin, is sent home with a written statement of the reason for so doing, and in the case of the eruptive diseases and diphtheria, reports are immediately sent to the district medical inspectors for inspection and supervision—medical inspectors, not mere inspectors of nuisances. The advantages of this system are obvious. During the three months (65 schools days) in which the system has been in operation, 63,812 children were thus examined, and from among them 4,183 were excluded from school for one reason or another.

Dr. Hermann Biggs, in detailing the proceedings of the sanitary authorities of the city of New York, says that they possess the most autocratic powers, and confesses that the measures they are prepared to introduce and enforce might seem radical and arbitrary if they were not plainly designed for the public good. But he maintains that "even among the most ignorant of our foreign-born population few, or no, indications of opposition or resentment are exhibited to the exercise of arbitrary power in sanitary matters." This he attributes to the fact that the government of the United States is democratic. Be this as it may, one cannot but admit the logical completeness of the system which is enforced. Knowledge is of but small benefit unless it is practically applied.

